# Determination of serum adenosine deaminase and xanthine oxidase activity in Kangal dogs with maternal cannibalism

**DOI:** 10.14202/vetworld.2017.1343-1346

**Published:** 2017-11-15

**Authors:** N. Ercan, M. Koçkaya, S. Kapancik, D. Bakir

**Affiliations:** 1 Department of Biochemistry, Faculty of Veterinary Medicine, Cumhuriyet University, Sivas, Turkey; 2 Department of Physiology, Faculty of Veterinary Medicine, Cumhuriyet University, Sivas, Turkey; 3 Department of Biochemistry, Faculty of Medicine, Cumhuriyet University, Sivas, Turkey

**Keywords:** adeosine deaminase, Kangal dog, maternal cannibalism

## Abstract

**Aim:**

Kangal dogs, known as guard dogs in many countries of the world, have been found to eat their own puppies during their first 24 h following birth, which is called as maternal cannibalism. Adenosine deaminase (ADA) and xanthine oxidase (XO) are important enzymes for purine metabolism. In this study, the aim is to evaluate ADA and XO activities in Kangal dogs with maternal cannibalism.

**Materials and Methods:**

The material of the study consists of the blood sera of Kangal dog breed with and without maternal cannibalism in the breeders around Sivas city and its districts. ADA and XO activities in blood serum of these animals were investigated by spectrophotometric method.

**Results:**

ADA activities in Kangal dogs with maternal cannibalism were increased to the control group without maternal cannibalism (p<0.01).

**Conclusion:**

Postnatal measurement of ADA activity in dogs may be useful in assessing maternal cannibalism.

## Introduction

Maternal cannibalism, in which a mother eats her young after birth, is seen in various animal species ranging from insects to mammals and is often explained by nutritional deficiencies or environmental stress. It is rare in non-human primates and is considered an abnormal behavior observed only in high-stress conditions [1-4].

Adenosine deaminase (ADA) and xanthine oxidase (XO) are important enzymes for purine metabolism. The ADA enzyme plays a role in the catabolic pathway of purine. ADA catalyses the reaction in which deoxyadenosine is converted to deoxyinosine. Like other enzymes involved in the purine catabolic pathway, ADA is also commonly found in mammalian tissues. An important part of the ADA present in the serum is provided by lymphocytes. Thus, increased ADA activity is indicative of increased lymphocyte activation. ADA activity in T-cells is much higher than in B-lymphocytes. The ADA enzyme is capable of fulfilling the functions of T-lymphocytes and has a vital role in the division and multiplication and differentiation of these cells. For this reason, this enzyme is described as a marker for cellular immunity. It is associated with many inflammatory diseases because it mediates the regulation of the immune response [5-7].

ADA is a cytoplasmic enzyme required for the differentiation and proliferation of lymphocytes. The increase in serum ADA activities is due to increased immunity. Decreased values are associated with immune deficiency. In dogs and pets, ADA has been described as a marker in many chronic diseases, such as visceral leishmaniasis, ehrlichiosis, brucellosis, and leukemia, and with an increase in parasitic conditions [8-10]. It has also been shown that people with unhealthy gestation show an increase in the incidence of major depression and postpartum depression [11-14].

ADA is considered to be an important marker in cell-mediated immunity [7]. It is associated with cellular immunity suppressed in pregnancy. Hence, ADA activity can change after pregnancy. Some studies have identified the activities of ADA activity increase [15,16], and other studies have shown that ADA activity falls during immunosuppressive situations such as pregnancy [17].

The XO enzyme is a member of the molybdenum iron-sulfur flavin hydroxylases. This enzyme was first discovered in milk [18]. XO enzyme is widely found in all tissues such as the liver, lung, heart, intestine, kidney, brain, and blood [19,20]. The most important enzymatic source of superoxide anion, which plays a role in the pathogenesis of many diseases, is XO enzyme. Therefore, changes in the activity of XO enzyme have been linked to neurological diseases [11,21].

In this study, it is aimed to determine ADA and XO activities, which are involved in purine catabolism and are the speed-limiting enzyme of this path, in the sera of Kangal dogs with maternal cannibalism and to evaluate the effects of ADA and XO on maternal cannibalism.

## Materials and Methods

### Ethical approval

This study was carried out with the consent no: 31/25.01.2016 of Local Ethics Committee of Animal Experiment, Cumhuriyet University.

### Sample collection

Ten maternal Kangal dogs, who have the same feeding and accommodation conditions, are physically healthy and show maternal cannibalism behaviors in the first 24 h after birth were included in the study, while 10 maternal Kangal dogs without maternal cannibalism were included in the study as a control group.

Blood samples were obtained from maternal cannibalism and control groups and then centrifuged at 4000 rpm for 10 min to obtain blood sera. These serum samples were stored at −80°C until the day of analysis.

### Measurement of serum ADA activity

Serum ADA activity was evaluated spectrophotometrically according to a method of Giuisti (1974) which is based on the formation ammonia, produced when ADA acts in excess of adenosine [22]. Results were meaned as units per liter (U/L).

### Measurement of serum XO activity

Serum XO activity was determined as spectrophotometrically according to Worthington (1972) by the consist of uric acid from xanthine by means of increase in Worthington-Enzyme [23]. Results were meaned as units per milliliter (U/ml).

### Statistical analysis

Data were assessed using the Student’s t-test, and intergroup comparisons were performed using the SPSS package program [24].

## Results

ADA and XO activities were determined in Kangal dogs with or without maternal cannibalism in this study (Table-1).

**Table-1:** Activities of ADA and XO between groups.

Parameters	Maternal cannibalism	Control	p value
ADA (U/L)	2.62±0.64	0.58±0.09	0.009
XO (U/L)	0.25±0.02	0.32±0.03	0.135

n=10; mean±SE. SE=Standard error, ADA=Adenosine deaminase, XO=Xanthine oxidase

When ADA activities were compared between maternal cannibalism and control group, maternal cannibalism group activity was statistically higher than the control group (p<0.01), but there was no significant difference in XO activities (Table-1). There was no correlation between ADA and XO activities.

## Discussion

The incidence of cannibalism has been examined in a series of developmental surveys of hamsters manipulated during the prenatal period by the idea that female hamsters kill their own offspring as part of an organized mechanism with the need for metabolic energy. In Experiment 1, more cannibalism was seen when the females were fed with lightweight and simpler diets. In Experiment 2, the food restriction (FR) during breastfeeding increased the activity of cannibalism in mild, less fatty mothers. These results show that an important factor influencing pupal cannibalism is the general presence of metabolic fuels from both external (food supply) and internal (fat tissue) sources [25]. In a study conducted by Ricci *et al*. [26], various results of maternal and fetal activities of maternal FR during pregnancy were examined. At maternal activity, increased frequency of cannibalism has been observed after maternal FR. In this study, maternal Kangal dogs, from whom blood samples were received, were fed as *ad libitum* without any FR in both groups.

Pregnancy and ADA are both associated with suppression of cellular immunity. Oladipo *et al*. [14] were found that mean serum ADA activities were found to be higher in non-pregnant women than in normal pregnant women (p<0.001). Among the pregnant women, mean of serum ADA was significantly higher in hypertensive and preeclamptic women than in normal pregnant group (p<0.001), indicating a possible decrease in cellular immunity in normal pregnancy and increased preeclampsia cell-mediated immunity.

Alan *et al*. [17] evaluated ADA activities in pregnancy and postpartum period at sheep. The ADA activities at high levels were obtained on the day of mating and postpartum, but it is observed that it started to decline as the pregnancy period progressed, and therefore, the lowest values were obtained at the period closest to the birth.

In another study, nitric oxide activities and ADA activities were determined in plasma and amniotic fluid in healthy pregnant (10) and non-pregnant (10) sheep. Plasma ADA activity was higher in healthy pregnant sheep (p<0.001) [15].

Corticotropin-releasing hormone (CRH) is released by stress factors and produces stress-induced behavioral effects. In a study investigating the behavioral responses to stress and the effects of stressors on maternal behavior, the effects of CRH (0.5-4 μg) intracerebroventricular infusion on behavior toward the offspring were examined in rats with maternal experience. Higher doses of CRH (1-4 μg) have shown to significantly increase killing offspring in naive rats [27]. Pedersen *et al*. [27] found that the formation of maternal cannibalism was stress induced by exogenously giving CRH. In this study, increased ADA activity may be associated with maternal cannibalism as a stress-induced behavior in Kangal dogs.

## Conclusion

In this study, the fact that the decrease in ADA activity in dogs without maternal cannibalism was due to the immunosuppressive nature of the pregnancy in relation to the normal gestation period was determined. However, the remarkable increase in ADA activity is noteworthy in maternal cannibalism and in maternal dogs who eat their offspring following birth. There has been no FR and no stress factors in the environmental or accommodation conditions, which may lead to an increase in this behavior, and they are physically healthy dogs. In these dogs, serum ADA activity can be described as an oxidative stress-induced condition in the postpartum.

It is important that the maternal cannibalism, which appears in the world famous guard and companion Kangal dogs, is assessed and is detected before it arises. ADA activity may have a pathophysiological role in maternal cannibalism. It may predict prognosis and be used to select these dogs before it has happened.

## Authors’ Contributions

All authors designed this research. NE, MK, SE, and DB designed the experiment. MK collected field samples. NE, SK, and DB conducted the laboratory testing. NE and SK prepared the data sets. NE drafted the manuscript. All authors read and approved the manuscript.
